# A (poly)phenol-rich diet reduces serum and faecal calprotectin in older adults with increased intestinal permeability: the MaPLE randomised controlled trial

**DOI:** 10.1186/s12877-024-05272-y

**Published:** 2024-08-24

**Authors:** Mirko Marino, Cristian Del Bo’, Daniela Martini, Simone Perna, Marisa Porrini, Antonio Cherubini, Giorgio Gargari, Tomás Meroño, Nicole Hidalgo-Liberona, Cristina Andres-Lacueva, Paul A Kroon, Simone Guglielmetti, Patrizia Riso

**Affiliations:** 1 Department of Food, Environmental and Nutritional Sciences (DeFENS), Division of Human Nutrition, Università degli Studi di Milano, Milano, Italy; 2 Geriatria, Accettazione Geriatrica e Centro di Ricerca per l’Invecchiamento, IRCCS INRCA, Ancona, Italy; 3 Department of Clinical and Experimental Medicine, Università Politecnica delle Marche, Ancona, Italy; 4 Department of Food, Environmental and Nutritional Sciences (DeFENS), Division of Food Microbiology and Bioprocesses, Università degli Studi di Milano, Milano, Italy; 5 Biomarkers and Nutrimetabolomics Laboratory, Department of Nutrition, Food Sciences and Gastronomy, Faculty of Pharmacy and Food Sciences, CiberFES, ISCIII, University of Barcelona, Barcelona, 08028 Spain; 6 Quadram Institute Bioscience, Norwich Research Park, Norwich, NR4 7UQ UK; 7 Department of Biotechnology and Biosciences (BtBs), Università degli Studi di Milano-Bicocca, Piazza della Scienza 4, Milano, Italy

**Keywords:** Ageing, Leaky gut, Inflammation, Calprotectin, Flavonoids, Phenolics, Diet

## Abstract

**Background:**

Older subjects are at risk of elevated intestinal permeability (IP) which can lead to immune system activation and low-grade systemic inflammation. Dietary changes are a potential strategy to reduce IP. The MaPLE project evaluated the hypothesis that increasing (poly)phenol intake would beneficially impact on several important markers and pathways related to IP. The objective of the present study was to assess the effects of the MaPLE (poly)phenol-rich diet (PR-diet) on additional IP-related biomarkers and any relationships between biomarker responses.

**Methods:**

A randomised, controlled, crossover study was performed involving 51 participants (≥ 60 y) with increased IP, as determined by serum zonulin levels. Participants were randomly assigned to one of two intervention groups: a control diet (C-diet) or a PR-diet. Each intervention lasted 8 weeks and was separated by an 8-week washout period. For the present study, serum and faecal samples were used to measure zonula occludens-1 (ZO-1), occludin, adiponectin, calprotectin, faecal calprotectin, soluble cluster of differentiation 14 (sCD14), interleukin-6 receptor (IL-6R), and vascular endothelial-cadherin (VEC) levels using quantitative ELISA assays. Data were analysed using ANOVA, and Spearman and network correlation analysis were performed to identify the relationship among biomarkers at baseline.

**Results:**

Among the different markers analysed, a significant reduction was observed for faecal and serum calprotectin (*p* = 0.0378 and *p* = 0.0186, respectively) following the PR-diet, while a significant increase in ZO-1 was found (*p* = 0.001) after both the intervention periods (PR-diet and C-diet). In addition, a time effect was observed for VEC levels showing a reduction (*p* = 0.038) following the PR-diet. Based on network correlation analysis, two clusters of correlations were identified: one cluster with high levels of serum calprotectin, faecal calprotectin, sCD14, interleukin (IL)-6, tumor necrosis factor (TNF)-α, C-reactive protein (CRP) and bacterial DNAemia (16 S rRNA gene copies), with potential inflammatory-induced intestinal permeability. Differently, the other cluster had high levels of serum occludin, IL-6R, soluble intercellular adhesion molecule-1 (sICAM-1) and VEC, with potential inflammatory-induced endothelial dysfunction.

**Conclusions:**

Overall, this study provides further support to the hypothesis that a (poly)phenol-rich diet may help to ameliorate intestinal permeability-associated conditions. In this regard, calprotectin might represent a promising biomarker since it is a protein that typically increases with age and it is considered indicative of intestinal and systemic inflammation. Further research is needed to develop targeted (poly)phenol-rich diets against age-related gut dysfunction and inflammation.

**Trial registration:**

28/04/2017; ISRCTN10214981; 10.1186/ISRCTN10214981.

## Background

The intestinal barrier (IB) serves as a crucial physical and functional barrier, maintaining separation between the external environment and the systemic circulation. It plays a vital role in various essential functions, such as controlling the selective uptake of numerous substances [1]. The transport of molecules across the intestinal epithelium occurs through two major pathways: transcellular and paracellular routes. The transcellular route involves carrier-mediated transport facilitated by transporters and channels, while the paracellular route relies on diffusion and is not carrier-mediated [2]. An intact intestinal barrier (IB) is crucial for preventing the entry of pathogens, toxins, antigens, and pro-inflammatory agents [3]. This barrier is regulated by specialized transmembrane proteins called tight junctions (TJs) that are found at the connections between epithelial cells. These TJs play a vital role in controlling intestinal permeability (IP), which governs the exchange of molecules, such as solutes and fluids, between the intestinal lumen and the bloodstream [4]. When TJs are disrupted, paracellular permeability increases, allowing the passage of molecules via an unregulated diffusion process even if their molecular weight exceeds 150 Da [5]. This disruption results in a “leaky” intestine and an elevation in IP.

In this regard, emerging evidence suggests that aging might be associated with increased IP, contributing to the development of chronic low-grade inflammation, also known as inflammaging. In particular, pro-inflammatory molecules permeate from the intestinal lumen to the mucosal layer and eventually enter the bloodstream, triggering the activation of the mucosal immune system. Prolonged activation of the immune system leads to chronic inflammation, tissue damage, and subsequent systemic inflammatory responses, which can give rise to various age-related pathologies, including metabolic disorders, and cardiovascular disease [6]. However, the evaluation of IP in humans can be challenging for several reasons. Direct measure of IP, which involves the use of biopsies, is highly accurate but invasive and not applicable for routine clinical use [7]. Non-invasive techniques are preferred for assessing IP in humans. For example, the measurement of lactulose and mannitol excretion in urine after their oral administration as a test solution can indicate changes in IP. This approach relies on the differential molecular size of lactulose and mannitol to assess permeability. However, it is noteworthy that the method lacks specificity, as it can be influenced by factors like renal clearance and should be interpreted cautiously [8]. Also, blood tests can be used to assess the levels of certain biomarkers like zonulin and lipopolysaccharide (LPS), which are associated with IP [9]. However, these biomarkers may not be exclusive to IP, making the interpretation challenging. Thus, there is specific interest in promoting research able to identify one or a set of markers potentially useful to target IP and related conditions and to verify the impact of intervention strategies.

Within this framework, diet could have a profound impact on gut function and overall gastrointestinal health due to a combination of various food components, including macronutrients, micronutrients, fibre, and bioactive compounds [10–12]. It has been reported that specific dietary patterns, such as the Western diet characterised by a high intake of processed foods, red meat, and sugary beverages, are linked to an increased risk of developing gastrointestinal disorders, including inflammatory bowel disease (IBD), irritable bowel syndrome (IBS), and colorectal cancer [13, 14]. In contrast, a balanced and diverse diet rich in food bioactives contributes to optimal gut function [15]. For instance, (poly)phenols have been proposed as potential modulators of IP. (Poly)phenols are a diverse group of plant-derived compounds that have been shown to exert antioxidant, anti-inflammatory, and immunomodulatory properties at both intestinal and systemic levels [16]. (Poly)phenols could act on various aspects of the intestinal barrier, both directly and indirectly. These include regulating tight junction function, modulating the production of inflammatory cytokines, and activating antioxidant genes [17]. Additionally, (poly)phenols are significantly metabolised by the gut microbiota, thus affecting the intestinal microbial ecosystem. Despite increasing evidence supporting the role of (poly)phenols in regulating IP, the precise mechanisms of action remain not completely understood. Moreover, there is a scarcity of human intervention studies that have specifically investigated the impact of (poly)phenols on IP. Additionally, although novel biomarkers for assessing IP have been proposed, there is still limited understanding of the roles of these molecules within the context of intestinal physiopathology thus posing challenges in interpreting the study findings.

In the context of the MaPLE (Microbiome mAnipulation through Polyphenols for managing Leakiness in the Elderly) project, we have obtained significant findings that underline the complexity of evaluating intestinal permeability (IP) and the associated challenges. Our randomised controlled crossover intervention with a (poly)phenol-rich diet (PR-diet) demonstrated a reduction in serum zonulin levels, particularly in women and in subjects with high initial zonulin levels, who also experienced reductions in diastolic blood pressure and blood glucose levels [18]. Additionally, we observed a negative correlation between fiber-fermenting and butyrate-producing bacteria and markers of inflammation, indicating a link between gut bacterial composition and inflammatory status [18]. The PR-diet also significantly increased these beneficial bacteria, reinforcing the interaction between fiber, (poly)phenols, and gut microbiota activity [18]. Moreover, metabolites produced following the PR-diet intervention were inversely associated with serum zonulin levels, and we noted differences in (poly)phenol metabolism based on serum zonulin levels [19, 20]. Further, our findings revealed a positive association between serum zonulin and blood DNAemia, suggesting that paracellular permeability of epithelial and endothelial cell layers may facilitate bacterial translocation into the bloodstream [21]. The PR-diet intervention significantly reduced serum zonulin and interleukin-6 levels in subjects with higher blood DNAemia, with trends towards reductions in bacterial DNAemia, BMI, and TC/HDL ratio [22]. Collectively, these results indicate that a PR-diet may be a promising approach for managing IP, inflammation, and gut function.

Given the complexity highlighted by these results, it becomes evident that evaluating IP is multifaceted and requires additional biomarkers to better validate the effects of a (poly)phenol-rich dietary pattern. There is a pressing need to identify a panel of biomarkers that can effectively cluster subjects and more accurately reflect their intestinal health. Such an approach will not only enhance our understanding of the role of PR-diets in managing IP but also contribute to the development of reliable and accurate biomarkers for IP assessment, potentially serving as alternatives to the current gold standard methodologies when they are not applicable.

Therefore, the objective of this study was twofold. First, to assess the impact of the diet rich in (poly)phenols on selected biomarkers chosen due to their potential involvement in IP. Second, to identify potential clusters of markers that could function as indicators of physiological perturbations associated with IP in the older population.

## Methods

### Study design, subject selection, and characteristics of participants

Detailed information about the study design, subject selection and the characteristics of the participants is reported in Guglielmetti et al., [23] and Del Bo’ et al., [18]. Briefly, a total of 51 older subjects were enrolled at Civitas Vitae (OIC Foundation, Padua, Italy). The selection of subjects was carried out in collaboration with physicians and staff at Civitas Vitae [23]. Older subjects (age ≥ 60 years) with increased IP (evaluated by serum zonulin levels), a favourable nutritional and cognitive status, no current history of diabetes, heart disease and gastrointestinal conditions, including gastrointestinal intolerances and disorders, renal insufficiency, incontinence, and not taking antibiotics or anti-inflammatory drugs (in the last month before the intervention period) were selected and included in the study. The study was an 8-week randomised, repeated-measure crossover intervention trial comparing a (poly)phenol-rich (PR) diet versus a control (C) diet. The dietary intervention protocol involved substituting low-(poly)phenol products in the control diet with PR-foods, based on the nutrient composition and total (poly)phenol content of the participants’ daily menu. The PR-foods included berries and related products, blood orange and juice, pomegranate juice, green tea, Renetta apple and purée, as well as dark chocolate (callets and cocoa powder-based drink). The PR-diet was designed to approximately double the estimated total (poly)phenol intake compared to the control diet. The PR-foods provided an average of 724 mg of total (poly)phenols per day, as previously reported [18]. During the PR-dietary intervention, participants received three servings of selected PR-foods per day. Further details about the study design and the diet composition are reported in Guglielmetti et al. [23] and Martini et al. [24].

At the beginning and at the end of each intervention arm (C-diet vs. PR-diet), blood and faecal samples were collected for the analysis of the different markers as previously reported [18]. Briefly, following an overnight fast, blood samples were collected in Vacutainer tubes with silicone gel to separate the serum and kept at room temperature for at least 30 min. The serum was then isolated by centrifuging the tubes at 1400 g for 15 min at 4 °C. Fecal samples were collected using specific containers designed for this purpose. Serum and fecal samples were divided into small aliquots, placed into specific vials, and stored at -80 °C until further analysis.

The following study received approval by the Ethics Committee of the Università degli Studi di Milano (15/02/2016; ref.: 6/16/CE_15.02.16_Verbale_All-7) and registered at ISRCTN registry (ISRCTN10214981). Written consent was obtained from all participants.

### Evaluation of clinical and functional parameters

The comprehensive details of measuring clinical and functional biomarkers in blood samples have been previously reported [18]. In summary, fasting serum levels of glucose, insulin, creatinine, uric acid, aspartate aminotransferase (AST), alanine aminotransferase (ALT), gamma-glutamyl transferase (GGT), total cholesterol (TC), high-density lipoprotein cholesterol (HDL-C), low-density lipoprotein cholesterol (LDL-C), and triglycerides (TG) were assessed using routine biochemical analysis. Zonulin, C-reactive protein (CRP), tumor necrosis factor-α (TNF-α), and IL-6 were measured using ELISA kits (IDK^®^ Immundiagnostik; R&D Systems—cat#DCRP00, 150 HSTA00E, HS600B—respectively). The concentration of intercellular adhesion molecule-1 (ICAM-1) and vascular cell adhesion molecule-1 (VCAM-1) in serum samples was determined using an ELISA kit (Booster^®^, Vinci Biochem S.r.l.). The HOmeostasis Model Assessment (HOMA) and Cockroft-Gault (CG) indexes were calculated based on previously defined formulas in the literature [25]. DNA damage was detected in peripheral blood mononuclear cells (PBMCs) as formamidopyrimidine DNA glycosylase (FPG)-sensitive sites and DNA resistance to H_2_O_2_-induced oxidative stress by comet assay [26]. Quantification analysis of blood bacterial DNA was performed by Vaiomer SAS (Labège, France) as previously reported [22].

### Analysis of additional biomarkers

Serum was obtained after blood centrifugation for 15 min at 2300 X g at 4 °C. Two aliquots of the sample (200 µL) were split into 3 Eppendorf tubes and stored at -80 °C until analysis. In the present study serum and faecal samples were used to measure zonula occludens-1 (ZO-1; cat# MBS2605490), occludin (cat# MBS761051), adiponectin (cat#AG-45 A-0001YEK-KI01), calprotectin (cat# HK379), faecal calprotectin (cat# HK382), soluble cluster of differentiation 14 (sCD14; cat# HK320), interleukin-6 receptor (IL-6R; cat# AB181424), human LPS binding protein (LBP; cat# AB279407), and vascular endothelial-cadherin (VEC; cat#EK1317) levels through ELISA kits. The assay is based on the method of competitive ELISA consisting in the addition to each sample (including standard and control samples) of a biotinylated tracer (at the first step) and the use of a pre-coated 96-well plate with polyclonal antibody. The peroxidase-labeled streptavidin addition is used to bind the biotinylated tracer. After the reaction, the plate reader TECAN Infinite F200 (Tecan Group Ltd. Mannedorf, Switzerland) was used to read the fluorescence at 450 nm. The concentration of serum biomarkers was calculated by a 4PL standard curve as reported by the manufacturer.

### Statistical analysis

The normal distribution of the variables was checked using the Kolmogorov-Smirnov test and using Q-Q graphs. Descriptive statistics representing raw data for each group (control and treatment) and the full sample were provided, including median, interquartile range and frequencies, where appropriate.

Non-normally distributed data were analyzed using the Wilcoxon matched-pairs signed rank test to assess differences within groups. For each subject, we calculated the change (delta) between measurements in the two periods for each treatment. When the variable was normally distributed, paired t-test was used and the mean of differences was reported instead of the median of differences. Due to the small sample sizes and non-normally distributed data, the Friedman test was utilized to corroborate the statistical significance observed through the Wilcoxon matched-pairs signed rank test on intra-group deltas, to determine if there are significant differences in the responses between the various treatments. If significant differences were found, an additional post-hoc test Dunn’s multiple comparisons test has been performed to identify which specific treatments are different from each other. Variances were statistically significant for p-value < 0.05. All analyses were performed using GraphPad Prism version 8.0.2 for Windows, GraphPad Software, (Boston, Massachusetts USA).

A two-dimensional visualisation technique “heatmap” analysis has been applied to the Spearman correlation matrix using JASP software. (JASP Team, 2019; jasp-stats.org). A Network correlation analysis has been used considering the two main clusters related to intestinal permeability and vascular function (JASP Team, 2019; jasp-stats.org).

## Results

### Baseline characteristics of the participants

The age of the 51 subjects (29 females and 22 males) enrolled ranged between 60 and 98 years with a mean of 78 ± 10 y. The clinical characteristics of the study participants at baseline, the consumption of both macro and micronutrients throughout the PR and control diet periods, and the key findings from the trial, have previously been published [18].

### Effect of the dietary intervention on the biomarkers under study

**Fig. 1:**
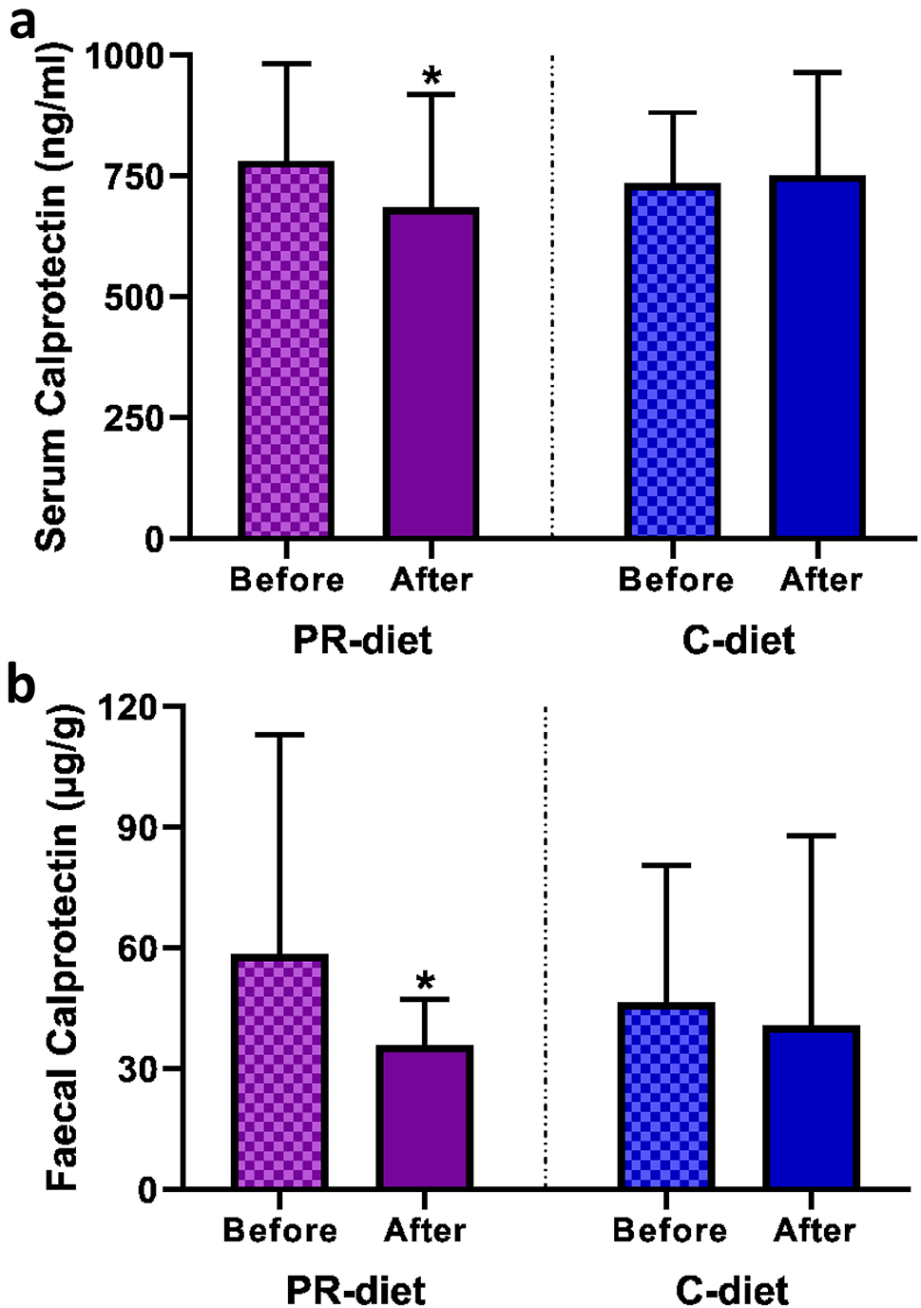
Effect of the dietary intervention on serum (**a**) and faecal (**b**) calprotectin. Data are expressed as the median and interquartile range (IQR). Differences within groups were assessed using the Wilcoxon matched-pairs signed rank test. Data with asterisk are significantly different (*p* < 0.05). PR-diet, (poly)phenol-rich diet; C-diet, control diet

Table 1 reports the values of biomarkers related to IP and inflammation at baseline and following the dietary intervention. A statistically significant effect was observed for faecal (*p* = 0.0378) and serum calprotectin levels (*p* = 0.0186), which decreased after the PR-diet (Fig. 1a and b). Additionally, a statistically significant increase in ZO-1 was found (*p* = 0.001) after both the PR-diet and C-diet. A time effect has been observed for vascular endothelial (VE)-cadherin showing a reduction following the PR-diet (*p* = 0.038). Conversely, sCD14, occludin, adiponectin and IL-6R were not significantly modified by the dietary intervention.

**Table 1:** Effect of 8-week intervention with PR-diet and C-diet on biomarkers under study

Variables (*n* = 51)	Before PR-diet median (IQR)	After PR-diet median (IQR)	Median change PR group (95% CI)	Before C-diet median (IQR)	After C-diet median (IQR)	Median change C group (95% CI)	Median difference between groups	*p*-value within PR group	*p*-value within C group	*p*-value between groups
Calprotectin (ng/mL)	782.20 (595.05)	684.80 (619.80)	-101.6 “-155.2 to -57.10”	735.00 (572.95)	751.90 (566.20)	-17.90 “-100.4 to 41.80”	-104.1 “-227.6 to 2.400”	**0.0072**	> 0.9999	**0.0186** ^**#**^
Faecal Calprotectin (µg/g)	58.48 (115.45)	35.94 (65.74)	-12.48 “-30.79 to -3.708”	46.58 (119.01)	40.78 (114.41)	-3.004 “-10.70 to 6.620”	-9.240 “-22.58 to -0.7620”	**0.0007**	0.5929	**0.0378** ^**#**^
sCD14 (µg/mL)	2.41 (0.77)	2.38 (0.75)	-0.02000 “-0.1000 to 0.07000”	2.36 (0.74)	2.42 (0.72)	0.000 (-0.7000 to 1.200)	0.02000 “-0.2300 to 0.09000”	0.2504	0.9453	0.2097
VE-cadherin* (ng/mL)	224.50 (123.45)	200.55 (116.90)	-11.98 “-25.25 to 1.296”	205.30 (121.42)	206.80 (112.30)	-5.425 “-21.24 to 10.39”	-6.684 “-30.01 to 16.64”	**0.0380**	0.4941	0.2837
ZO-1 (ng/mL)	1.36 (1.67)	1.68 (1.06)	0.5050 “0.2400 to 0.7600”	1.21 (1.24)	1.75 (1.33)	0.2700 “0.1200 to 0.7500”	0.1420 “-0.3590 to 0.3799”	**0.0052**	**0.0084**	0.4529
Occludin (ng/mL)	4.48 (2.27)	4.32 (2.55)	0.01000 “-0.06000 to 0.1500”	4.36 (2.70)	4.46 (2.96)	0.06000 “-0.05000 to 0.2500”	-0.01900 “-0.3000 to 0.1160”	0.3679	0.1333	0.1018
Adiponectin (µg/mL)	5.93 (3.69)	5.33 (4.25)	-0.07000 “-0.2800 to 0.1600”	5.49 (4.13)	5.19 (4.46)	0.05000 “-0.2100 to 0.2300”	0.000 “-0.6700 to 0.2400”	0.1364	0.8314	0.1353
IL-6R (ng/mL)	23.89 (13.68)	23.34 (14.85)	0.7400 “-0.9400 to 2.040”	23.04 (15.99)	22.63 (13.02)	1.110 “-0.4200 to 2.260”	-0.5900 “-2.710 to 1.100”	0.5816	0.4357	0.3119
LBP (ng/mL)	9.96 (5.75)	9.61 (4.18)	0.3450 “-0.2800 to 1.140”	10.34 (6.04)	10.21 (5.47)	0.205 “-0.8300 to 1.050”	-0.4500 “-1.270 to 0.310”	0.1427	0.9828	0.1400

All data are expressed as median and interquartile range (IQR). Differences within groups were assessed using the Wilcoxon matched-pairs signed rank test. Data with asterisk are significantly different (*p* < 0.05). Values in 95% CI of the difference represent differences of the medians (Hodges-Lehmann estimate) for continuous variables

PR, (poly)phenol-rich diet; C, control diet; soluble cluster of differentiation 14 (sCD14); vascular endothelial-cadherin (VE-cadherin); zonula occludens-1 (ZO-1); interleukin-6 receptor (IL-6R); lipopolysaccharide-binding protein (LBP)

*Variable is normally distributed. Paired t-test has been used instead of the Wilcoxon matched-pairs signed rank test. Mean of differences has been reported instead of the median of differences

^**#**^Friedman test has been used to corroborate the Wilcoxon matched-pairs signed rank test and both tests are statistically significant (*p* < 0.05)

### Correlation heatmap among the different biomarkers at baseline

**Fig. 2:**
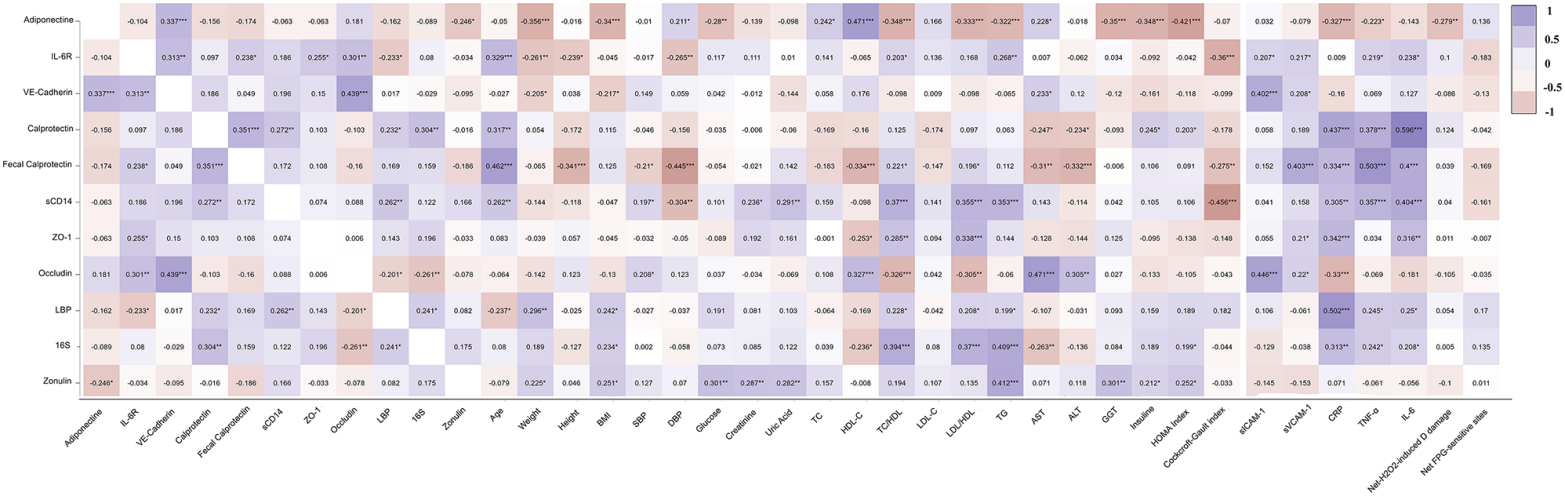
Correlation heatmap among the different biomarkers at baseline. The heatmap provides a visual representation of the correlation coefficients between all biomarkers, with colours denoting the strength of associations. Colour intensity within the cells corresponds to the magnitude of these relationships, with blue hues indicating positive correlations and red hues signifying negative correlations. The colour scale, positioned to the right of the panel, quantitatively conveys the degree of correlation. Each cell displays pair-wise Spearman correlation coefficients as a measure of association. Soluble cluster of differentiation 14 (sCD14); vascular endothelial-cadherin (VE-Cadherin); interleukin-6 (IL-6); interleukin-6 receptor (IL-6R); bacterial DNAemia (16 S); tumor necrosis factor-α (TNF-α); C-reactive protein (CRP); zonula occludens-1 (ZO-1); lipopolysaccharide-binding protein (LBP); body mass index (BMI); systolic blood pressure (SBP); diastolic blood pressure (DBP); total cholesterol (TC); low-density lipoprotein cholesterol (LDL-C); high-density lipoprotein cholesterol (HDL-C); triglycerides (TG); alanine transaminase (ALT), aspartate aminotransferase (AST), gamma-glutamyl transferase (GGT); soluble intercellular adhesion molecule-1 (sICAM-1); soluble vascular adhesion molecule-1 (sVCAM-1)

Figure 2 shows the heatmap correlation at baseline for all investigated outcomes within the MaPLE project. Inflammatory biomarkers such as sCD14, CRP, IL-6 and TNF-α were directly correlated with each other. Serum levels of sCD14 were also positively correlated with serum calprotectin, LBP, age, SBP, creatinine, uric acid, TC/HDL, LDL/HDL, TG. Anti-inflammatory biomarker adiponectin was inversely correlated with CRP, BMI and HOMA index. Furtherly, serum calprotectin levels were positively correlated with LBP and 16 S rRNA gene copies, two candidate biomarkers of IP. Circulating calprotectin was also directly correlated with the age of participants, insulin, HOMA index, CRP, IL-6, TNF-α and faecal calprotectin. Faecal calprotectin was positively and strongly correlated with inflammatory biomarkers (CRP, IL-6 and TNF-α), sVCAM-1 and age

### Network correlation analysis among biomarkers at baseline

**Fig. 3:**
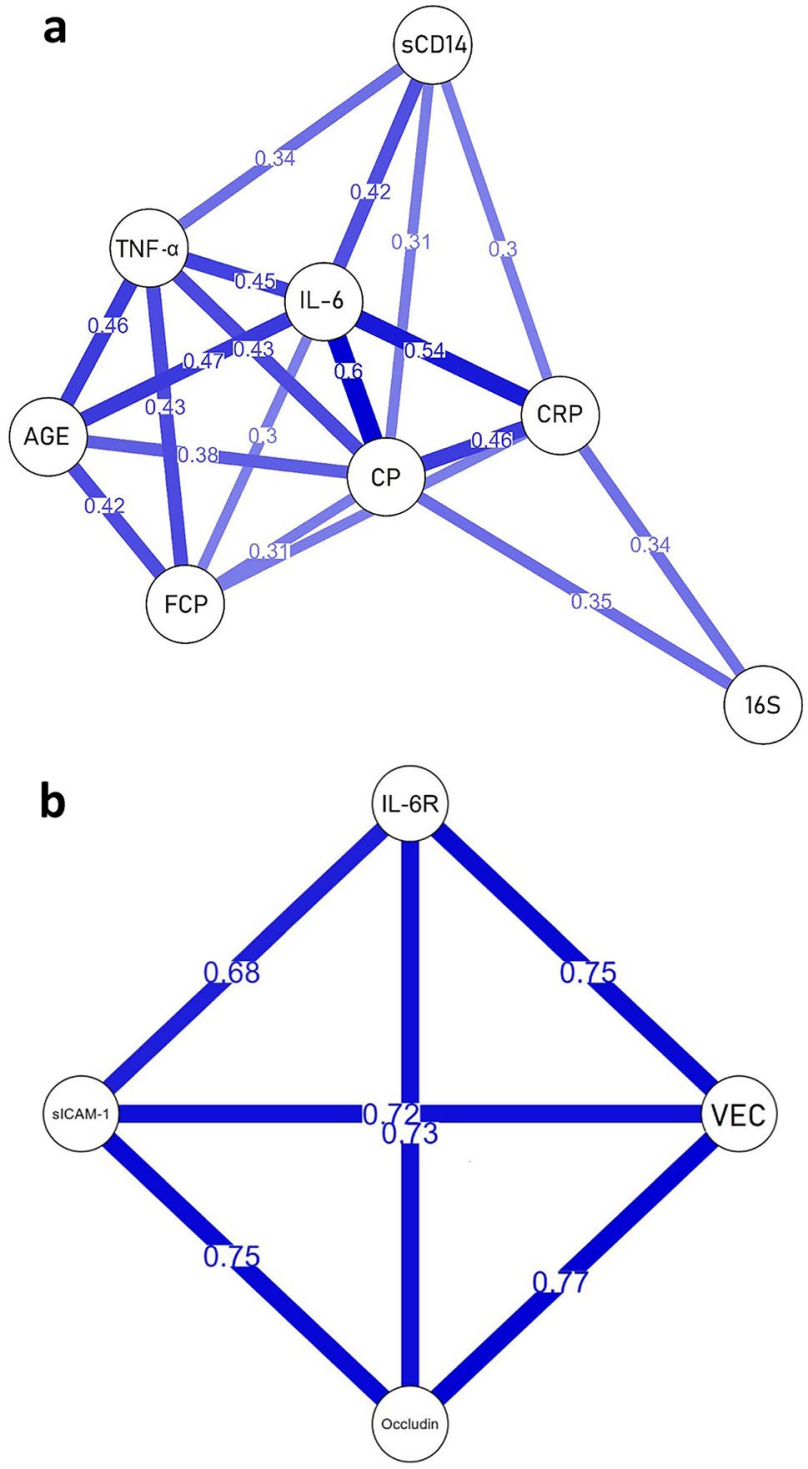
Network analysis of biomarker correlations at baseline. Network representation of relationship between the main panels of biomarkers statistically associated in Fig. 2. A panel of biomarkers related to inflammation and intestinal permeability (a) and a panel of biomarkers associated with vascular endothelial function (b). Blue lines represent positive associations and the number is the coefficient of correlation. Soluble cluster of differentiation 14 (sCD14); vascular endothelial-cadherin (VEC); interleukin-6 (IL-6); interleukin-6 receptor (IL-6R); calprotectin (CP); faecal calprotectin (FCP); bacterial DNAemia (16 S); soluble intercellular adhesion molecule-1 (sICAM-1); tumor necrosis factor-α (TNF-α); C-reactive protein (CRP)

Figure 3a and b show the network correlation analysis among the main panel of biomarkers statistically associated in Fig. 2. In particular, this panel consists of biomarkers of intestinal permeability and biomarkers correlated to them.

Figure 3a shows the network of biomarkers associated with neutrophil activation, inflammation and IP. Serum calprotectin levels show a pivotal role in the network, being positively correlated with all the biomarkers involved, such as faecal calprotectin, soluble cluster of differentiation 14 (sCD14), interleukin (IL)-6, tumor necrosis factor (TNF)-α, C-reactive protein (CRP), bacterial DNAemia (16 S rRNA gene copies) and age.

In Fig. 3b, the network of biomarkers associated with vascular endothelial function is shown. Specifically, this cluster of correlation has high levels of serum occludin, IL-6 receptor (IL-6R), soluble intercellular adhesion molecule-1 (sICAM-1) and vascular endothelial (VE)-cadherin, with potential inflammatory-induced endothelial dysfunction.

## Discussion

Our RCT demonstrated the efficacy of a (poly)phenol-rich diet in reducing a key biomarker of immune response and systemic inflammation such as serum calprotectin and a more specific biomarker of intestinal inflammation like faecal calprotectin in older subjects.

Although there are multiple sources of evidence to support the anti-inflammatory effects of (poly)phenols, their use in the context of IP has been almost exclusively studied in vitro or in animal models. In addition, no studies have been conducted in vivo evaluating the impact of phenolic compounds on serum calprotectin. Calprotectin, a protein secreted by neutrophils, is prominently released in response to inflammation. This protein holds significant importance in the innate immune response, serving as an indicator of immune system activation and systemic inflammation. Serum calprotectin has recently garnered increased interest as a practical blood-based biomarker for IBD, as various studies have demonstrated that its concentration in the blood is associated with faecal levels, which serve as a reliable indicator of IBD. For instance, Kalla et al. [27] employed multivariable logistic regression analysis to assess the serum calprotectin levels in a cohort of 156 patients, including 83 individuals with IBD and 73 non-IBD subjects. The findings revealed a significant positive correlation between serum and faecal calprotectin levels. Moreover, serum calprotectin exhibited superior predictive capabilities for diagnosing IBD compared to other commonly used biomarkers such as C-reactive protein (CRP) and albumin. Notably, serum calprotectin demonstrated its potential as a prognostic indicator by accurately predicting treatment escalation and/or the need for surgery in IBD patients, particularly those with Crohn’s disease (CD). Also, Meuwis and coworkers [28] demonstrated that the median serum calprotectin level in CD patients was significantly higher (8,892 ng/mL) than in healthy controls (1,318 ng/mL). Furthermore, the authors showed that, when combined with CRP and faecal calprotectin, serum calprotectin could serve as a valuable complementary biomarker for predicting relapse after infliximab withdrawal in CD patients.

Consistent with earlier research, our study has confirmed a positive association between serum and faecal calprotectin levels and we found to be both reduced following the intervention. In accordance with our findings, the open pilot trial of Biedermann and colleagues [29] showed a reduction of faecal calprotectin in 11 male and female individuals diagnosed with active mild-to-moderate ulcerative colitis (UC). In particular, the participants were administered a bilberry-anthocyanin supplement, specifically 160 g of bilberry preparation containing 840 mg of anthocyanin per day, over a 6-week intervention period. This was compared to a group receiving a control product without ACNs. The authors observed a reduction of 61% (from 778 to 305 µg/g), slightly higher compared to the 39% reduction (from 58.5 to 35.9 µg/g). The lower reduction we found is likely attributable to our study population characterised by lower baseline levels of faecal calprotectin compared to individuals with UC involved in the study by Biedermann et al. [29]. The intervention led also to an improvement in the mean Clinical Activity Index (CAI) score and a reduction in both the endoscopic Mayo score and complete Mayo score, reflecting positive changes in disease severity and overall UC activity. Conversely, Solch-Ottaiano and coworkers [30] did not observe a significant reduction of faecal calprotectin levels after an intervention with a high (poly)phenol cranberry beverage (953 mg per daily serving) in healthy obese adults. The lack of effect could be explained by the experimental design involving a treatment with aspirin to simulate the loss of barrier function in a study population with low levels of faecal calprotectin at baseline (7.24 µg/g with respect to 58.48 µg/g of our study subjects).

In a previous study, serum calprotectin correlated to different biomarkers linked to inflammatory processes and metabolic dysfunction such as the levels of CRP, leukocytes, LDL, PAI-1, resistin and visfatin, in line with our findings [31]. Indeed, we found that serum calprotectin was associated with a cluster of biomarkers related to IP, inflammatory response, and age. Thus, the inclusion of biomarkers such as bacterial DNAemia, faecal calprotectin, IL-6, sCD14, CRP, and TNF-a may provide a comprehensive examination of the immune and inflammatory pathways. These biomarkers are commonly studied in the context of intestinal health, inflammation, and aging, indicating a connection between systemic inflammation, gut barrier function, and the aging process. Together these biomarkers could be useful to investigate immune and inflammatory pathways in response to dietary interventions. In this study, we measured circulating bacterial DNA in terms of 16 S rRNA gene copies (bacterial DNAemia) as well as faecal and serum calprotectin. Our correlation analyses confirmed the validity of these biomarkers in evaluating intestinal permeability and systemic inflammation. The interconnections between bacterial DNAemia and calprotectin underscore the crucial influence of bacterial translocation on the inflammatory process and metabolic health, highlighting its potential as a target for therapeutic interventions. IL-6 is a pro-inflammatory cytokine that plays a role in immune regulation and has been associated with various age-related diseases. sCD14, a soluble form of the CD14 receptor, is involved in immune activation and has been linked to gut barrier dysfunction. CRP and TNF-α are inflammatory biomarkers that are elevated in response to infection or inflammation.

In our older subjects, we also found the presence of a cluster characterised by high levels of VE-cadherin, occludin, sICAM, and IL-6R. These biomarkers are associated with endothelial function, tight junction integrity, and immune regulation. Their correlation further strengthens the link between immune dysregulation, gut barrier disruption, and age-related perturbations. Occludin is a transmembrane protein that is a key component of tight junctions, which are intercellular junctions between epithelial and endothelial cells. Its primary role is to regulate the permeability of cellular barriers, including the blood-brain barrier and the intestinal barrier. As a biomarker, occludin can be indicative of the integrity and function of these cellular barriers. sICAM is a soluble form of intercellular adhesion molecule-1 (ICAM-1), a cell surface glycoprotein that is expressed in various cell types, including endothelial cells and immune cells. ICAM-1 plays a vital role in the adhesion of leukocytes to endothelial cells during inflammation, facilitating immune cell trafficking to sites of infection or tissue damage. The shedding of sICAM occurs when ICAM-1 is cleaved from the cell surface, and sICAM can be detected in the bloodstream. As a biomarker, sICAM levels can reflect immune activation and the presence of inflammation. Elevated sICAM levels have been associated with various inflammatory conditions, including rheumatoid arthritis, atherosclerosis, and other autoimmune diseases. VE-cadherin is a transmembrane protein that plays a crucial role in cell-cell adhesion and maintaining endothelial barrier integrity. It is predominantly expressed in endothelial cells that line blood vessels and lymphatic vessels. VE-cadherin’s role as a biomarker lies in its association with vascular health, endothelial function, and its involvement in various physiological and pathological processes. Measuring VE-cadherin levels or activity can offer valuable information about endothelial barrier function, vascular permeability, and inflammatory responses, making it relevant in understanding and managing various vascular-related conditions and diseases [32]. Interestingly, in our study, we found a reduction of VE-cadherin levels following the intervention with the PR-diet. However, this difference in VE-cadherin levels was attributed to an effect of time but not to the treatment. Although there are no other intervention studies that have evaluated the effect of (poly)phenols on this biomarker, the results of some in vitro studies [33, 34] have demonstrated a potential protective effect. Thus, we can speculate that a longer intervention duration or a larger sample size could be necessary to reveal a positive effect of the PR-diet on VE-cadherin levels. Conversely, an increase in ZO-1 serum levels in both intervention groups was documented. This increase could be explained by the fact that our study individuals represent a fragile population target. Nevertheless, the magnitude of the change was apparently negligible, its clinical significance remains uncertain due to the lack of data in the literature on ZO-1 levels in different conditions.

The findings of the current RCT suggest that the identified biomarker clusters, including serum calprotectin and related biomarkers, may serve as a panel of indicators reflecting individual physiological states. These biomarkers could potentially be used to identify at-risk subjects for age-related health complications and guide dietary interventions. Based on our results, some of these biomarkers could be modulated through a (poly)phenol-rich diet, possibly mitigating the adverse effects of aging on gut barrier function and systemic inflammation, thereby contributing to the promotion of healthier aging and reduced risk of age-related diseases. Despite serum and faecal calprotectin was found to be directly correlated and reduced by the intervention with the PR-diet, evaluating faecal calprotectin levels compared to serum calprotectin levels can provide different clinical insights. While elevated faecal calprotectin levels suggest ongoing inflammation in the gut and may help clinicians in determining the severity of GI inflammation and the efficacy of treatment in GI-related disorders, increased serum calprotectin levels indicate a systemic inflammatory response and may help identify inflammatory conditions affecting multiple organs or tissues beyond the GI tract. Both assessments could have distinct clinical applications and provide complementary information about inflammatory processes, depending on the focus of the investigation and the conditions under consideration. Furtherly, calprotectin stability, consistent assay reproducibility, and cost-effectiveness have established it as an invaluable tool for clinicians in supporting diagnostic and therapeutic decisions.

The study findings may hold significant implications for future research and dietary interventions. Firstly, they offer further evidence of the potential benefits of a diet rich in (poly)phenols in reducing immune activation, intestinal and systemic inflammation, which are common aspects of aging and age-related diseases. This underscores the importance of exploring dietary strategies for promoting healthy aging. Secondly, the observed correlation between calprotectin and markers related to IP, inflammatory response, and age supports a possible link between gut health, inflammation, and the aging process. This relationship opens up new paths for investigating the underlying mechanisms of these connections and the potential role of phenolic compounds in modulating gut barrier function and age-related inflammation. The development of biomarker panels that include calprotectin and other relevant markers could lead to a more comprehensive evaluation of immune dysregulation and gut health during the aging process.

Future research should aim to validate the current findings in a broader range of populations, encompassing individuals with specific health conditions or varying levels of inflammation and IP. In addition, long-term studies are essential to establish the long-lasting effects of a (poly)phenol-rich diet on calprotectin levels and its overall impact on health outcomes, but also to determine the most effective dose and type of (poly)phenols to enrich the diet for these purposes. Moreover, deeper investigations are necessary to shed light on the underlying mechanisms and to establish causal relationships between these biomarkers and age-related gut dysfunction and inflammation. By addressing these research gaps, we could better understand the potential role of (poly)phenols in promoting healthy aging and mitigating age-related health challenges.

## Conclusions

The results obtained strengthen the hypothesis that a (poly)phenol-rich diet could play an important role in counteracting the worsening of intestinal permeability alteration and related conditions in the context of inflammaging by also reducing the levels of calprotectin, a promising biomarker as it is a protein known to typically elevate with age, serving as an indicator of both intestinal and systemic inflammation. If further research confirms these findings, it could be also possible in the future to develop and promote targeted/personalised (poly)phenol-rich diets to increase protection against age-related gut dysfunction and inflammation. By promoting a healthier intestinal barrier, (poly)phenols may potentially contribute to overall well-being and reduce the risk of age-related diseases associated with inflammaging.

## Data Availability

The MaPLE trial has been formally registered with the International Standard Randomised Controlled Trial Number (ISRCTN) registry, bearing the code ISRCTN10214981. In accordance with the data management plan of the MaPLE project, the datasets utilized in the present study are accessible via the Dataverse repository at https://dataverse.unimi.it/dataverse/DNAemia-Zonulin (dataset: https://doi.org/10.13130/RD_UNIMI/KGCL3D).
